# Evaluation of the SARS-CoV-2 Antibody Response to the BNT162b2 Vaccine in Patients Undergoing Hemodialysis

**DOI:** 10.1001/jamanetworkopen.2021.23622

**Published:** 2021-09-02

**Authors:** Kevin Yau, Kento T. Abe, David Naimark, Matthew J. Oliver, Jeffrey Perl, Jerome A. Leis, Shelly Bolotin, Vanessa Tran, Sarah I. Mullin, Ellen Shadowitz, Anny Gonzalez, Tatjana Sukovic, Julie Garnham-Takaoka, Keelia Quinn de Launay, Alyson Takaoka, Sharon E. Straus, Allison J. McGeer, Christopher T. Chan, Karen Colwill, Anne-Claude Gingras, Michelle A. Hladunewich

**Affiliations:** 1 Division of Nephrology, Department of Medicine, Sunnybrook Health Sciences Centre, Toronto, Ontario, Canada; 2 Division of Nephrology, Department of Medicine, Unity Health Toronto, Toronto, Ontario, Canada; 3 Department of Molecular Genetics, University of Toronto, Toronto, Ontario, Canada; 4 Lunenfeld-Tanenbaum Research Institute at Mount Sinai Hospital, Sinai Health System, Toronto, Ontario, Canada; 5 Li Ka Shing Knowledge Institute, St Michael’s Hospital, Unity Health Toronto, Toronto, Ontario, Canada; 6 Division of Infectious Diseases, Sunnybrook Health Sciences Centre, Toronto, Ontario, Canada; 7 Public Health Ontario, Toronto, Ontario, Canada; 8 Dalla Lana School of Public Health, University of Toronto, Toronto, Ontario, Canada; 9 Department of Laboratory Medicine and Pathobiology, University of Toronto, Toronto, Ontario, Canada; 10 Sinai Health System, Toronto, Ontario, Canada; 11 Division of Nephrology, University Health Network, Toronto, Ontario, Canada

## Abstract

**Importance:**

Patients undergoing hemodialysis have a high mortality rate associated with COVID-19, and this patient population often has a poor response to vaccinations. Randomized clinical trials for COVID-19 vaccines included few patients with kidney disease; therefore, vaccine immunogenicity is uncertain in this population.

**Objective:**

To evaluate the SARS-CoV-2 antibody response in patients undergoing chronic hemodialysis following 1 vs 2 doses of BNT162b2 COVID-19 vaccination compared with health care workers serving as controls and convalescent serum.

**Design, Setting, and Participants:**

A prospective, single-center cohort study was conducted between February 2 and April 17, 2021, in Toronto, Ontario, Canada. Participants included 142 patients receiving in-center hemodialysis and 35 health care worker controls.

**Exposures:**

BNT162b2 (Pfizer-BioNTech) COVID-19 vaccine.

**Main Outcomes and Measures:**

SARS-CoV-2 IgG antibodies to the spike protein (anti-spike), receptor binding domain (anti-RBD), and nucleocapsid protein (anti-NP).

**Results:**

Among the 142 participants undergoing maintenance hemodialysis, 94 (66%) were men; median age was 72 (interquartile range, 62-79) years. SARS-CoV-2 IgG antibodies were measured in 66 patients receiving 1 vaccine dose following a public health policy change, 76 patients receiving 2 vaccine doses, and 35 health care workers receiving 2 vaccine doses. Detectable anti-NP suggestive of natural SARS-CoV-2 infection was detected in 15 of 142 (11%) patients at baseline, and only 3 patients had prior COVID-19 confirmed by reverse transcriptase polymerase chain reaction testing. Two additional patients contracted COVID-19 after receiving 2 doses of vaccine. In 66 patients receiving a single BNT162b2 dose, seroconversion occurred in 53 (80%) for anti-spike and 36 (55%) for anti-RBD by 28 days postdose, but a robust response, defined by reaching the median levels of antibodies in convalescent serum from COVID-19 survivors, was noted in only 15 patients (23%) for anti-spike and 4 (6%) for anti-RBD in convalescent serum from COVID-19 survivors. In patients receiving 2 doses of BNT162b2 vaccine, seroconversion occurred in 69 of 72 (96%) for anti-spike and 63 of 72 (88%) for anti-RBD by 2 weeks following the second dose and median convalescent serum levels were reached in 52 of 72 patients (72%) for anti-spike and 43 of 72 (60%) for anti-RBD. In contrast, all 35 health care workers exceeded the median level of anti-spike and anti-RBD found in convalescent serum 2 to 4 weeks after the second dose.

**Conclusions and Relevance:**

This study suggests poor immunogenicity 28 days following a single dose of BNT162b2 vaccine in the hemodialysis population, supporting adherence to recommended vaccination schedules and avoiding delay of the second dose in these at-risk individuals.

## Key Points

**Question:**

What is the serologic response to the BNT162b2 COVID-19 vaccine in patients undergoing hemodialysis?

**Findings:**

In this cohort study of 142 patients receiving hemodialysis, humoral response was compared in 66 patients sampled 28 days after receipt of 1 dose of vaccine with 76 patients who received 2 doses of vaccine sampled 14 days after the second dose. Among those receiving 1 dose, 6% had antireceptor binding domain response above the median level of convalescent serum vs 41% of those who received 2 doses at 1 week, increasing to 60% by 2 weeks.

**Meaning:**

The findings of this study suggest that, given that patients receiving hemodialysis appeared to exhibit a poor humoral response to a single dose of BNT162b2 vaccine, the second dose should not be delayed.

## Teaser

This cohort study examines the immune response of patients receiving maintenance hemodialysis to vaccination with 1 vs 2 doses of the BNT162b2 vaccine.

## Introduction

SARS-CoV-2 with resultant COVID-19 has resulted in a global pandemic. Among those most severely affected are patients receiving maintenance hemodialysis who must visit facilities at least thrice weekly for life-sustaining treatment resulting in a 5 times greater risk for infection than the general population.^1^ Despite adherence to public health guidance, outbreaks have occurred in dialysis units.^2^ Furthermore, patients receiving hemodialysis are at greater risk for severe COVID-19, with 63% of patients receiving chronic hemodialysis who contract COVID-19 requiring hospitalization and a case fatality rate of 29% in Ontario, Canada.^1^ Confirmatory data from the US Renal Data System found mortality among patients receiving hemodialysis in early 2020 was 16% to 37% higher than in 2017-2019.^3^

Patients receiving hemodialysis frequently have a diminished immune response to vaccination compared with the general population, as observed during hepatitis B vaccination.^4^ Studies of natural COVID-19 infection in patients receiving hemodialysis found waning antibody concentrations by 3 months, raising the possibility that patients receiving hemodialysis may not develop an adequate vaccination response.^5^ In addition, randomized clinical trials for the BNT162b2 vaccine included few patients with kidney disease.^6^ Therefore, data on vaccine immunogenicity are lacking in this high-risk population.

Two doses of BNT162b2 vaccine were administered 21 days apart in randomized clinical trials. However, owing to vaccine shortages, some countries, including the UK and Canada, have prioritized first-dose vaccination of the general population^7^ while delaying the second dose for up to 3 to 4 months, offering a natural experiment for comparison of 1 vs 2 doses. To investigate the humoral response conferred by COVID-19 vaccination in the hemodialysis population, we conducted a prospective observational cohort study measuring SARS-CoV-2 immunoglobulin G (IgG) antibody levels following 1 vs 2 doses of the vaccine.

## Methods

In-center patients aged 18 years or older receiving hemodialysis, including those with prior COVID-19, were eligible for this single-center, prospective cohort study to evaluate SARS-CoV-2 antibody response to the BNT162b2 COVID-19 vaccine (Pfizer-BioNTech). Recruitment of 142 participants occurred between February 2 and March 3, 2021, at Sunnybrook Health Sciences Centre, and the study was completed on April 17, 2021. A subset of patients (n = 76) received 2 doses of vaccine, with the second dose a mean of 21 days (range, 19-28) following the first dose, and 66 patients received a single vaccine dose due to a public health policy change. In the 2-dose group, baseline was prior to the second dose and antibody levels were determined weekly until 14 days after the second vaccine dose. In those receiving a single vaccine dose, antibody levels were measured at baseline before vaccination and 28 days following the first dose. A written questionnaire captured vaccination-related adverse events. Health care worker (HCW) controls received 2 doses of BNT162b2 vaccine with antibodies measured 2 to 4 weeks following the second dose. This study was approved by the Sunnybrook Health Sciences and Mount Sinai Hospital Research Ethics Board. Written informed consent was obtained from all participants. This study followed the Strengthening the Reporting of Observational Studies in Epidemiology (STROBE) reporting guideline for cohort studies.

Antibodies targeting the full-length spike protein (anti-spike) and its receptor binding domain (anti-RBD) measured humoral response to SARS-CoV-2 vaccination and/or natural infection; antibodies to the nucleocapsid protein (anti-NP) detected natural SARS-CoV-2 infection because this antigen is not targeted by the BNT162b2 vaccine. SARS-CoV-2 IgG antibodies were measured on a custom automated enzyme-linked immunosorbent assay platform; the sensitivity and specificity of each assay were determined by precision-recall analysis from pre-COVID-19–negative and convalescent controls.^8,9^ Antibody levels are reported as relative ratios to a synthetic standard included as a calibration curve on each assay plate. Thresholds for positivity (seroconversion) were determined by aggregating data from negative controls and calculating the mean ±3 SDs. Relative antibody levels were also compared with the median levels of convalescent serum obtained 21 to 115 days after symptom onset in patients with COVID-19; expression of vaccination-induced antibody levels to convalescent individuals helps to define correlates of protection.^10^

### Statistical Analysis

Baseline characteristics were compared using a *t* test for continuous variables and χ^2^ or Fisher exact test for categorical variables. The association between reactogenicity following the second vaccine dose and anti-spike or anti-RBD seroconversion was assessed by a χ^2^ test. Antibody relative ratios between patients undergoing hemodialysis receiving 2 doses of vaccine and HCWs were compared using the Mann-Whitney test. Given the nonnormal distribution of relative ratios, which was not mitigated by log transformation, we set ratios with seroconversion above or below the median convalescent level as binary outcomes and used logistic regression to assess age, sex, and vaccine reactogenicity. Vaccine reactogenicity was binary in the model based on the presence of any of the following symptoms within 14 days after the second vaccine dose: pain, redness, swelling, fever, chills, fatigue, nausea or vomiting, diarrhea, myalgia, and joint pain. Simple imputation with the mean value across participants was used to address missing covariate data. With 2-sided testing, *P* < .05 was considered significant for all statistical findings. All analyses were performed using SPSS, version 17 (IBM Corp), and the multivariate logistic regression was performed using R, version 4.0.4 (R Project for Statistical Computing).

## Results

Among 142 of 157 consenting patients (90%) receiving in-center hemodialysis, the median age was 72 (interquartile range, 62-79) years. Of these, 94 were men (66%) and 48 were women (34%) (Table 1). At baseline, 15 patients (11%) had detectable anti-NP and 3 patients (2%) had reverse transcriptase polymerase chain reaction–confirmed COVID-19, indicating asymptomatic or mildly symptomatic infection in 12 of 15 patients (80%), which was unexpected given the unit’s extensive screening protocols. Clinical characteristics of patients receiving 1 vs 2 vaccine doses were similar, but those with 1 dose were slightly younger (10 [15%] vs 9 [12%] aged ≤55 years) and less likely to have diabetes (26 [39%] vs 37 [49%]) or ischemic nephropathy (8 [12%] vs 19 [25%]) as the cause of kidney failure. This finding is not surprising because Canada prioritized 2-dose vaccination of older individuals in aggregate living settings. The HCW controls had a median age of 46 (interquartile range, 44-69) years, 33 of 35 (94%) were women, and 3 of 35 (9%) had prior COVID-19. Of 211 patients with convalescent serum, median age was 59 (interquartile range, 34-55) years, 115 were men (55%), and the sample included patients with mild (defined as not requiring hospitalization), moderate, and severe disease.

**Table 1.** Clinical Characteristics of 142 Patients Undergoing Hemodialysis Receiving BNT162b2 Vaccine

Characteristic	No. (%)			*P* value^a^
	Total (n = 142)	1 Dose (n = 66)	2 Doses (n = 76)	
Age, median (IQR), y	72 (62-79)	72 (59-76)	75 (64-82)	.04
Age group				
≤55 y	19 (13)	10 (15)	9 (12)	.41
>55 y	123 (87)	56 (85)	67 (88)	
Sex				
Female	48 (34)	18 (27)	30 (39)	.13
Male	94 (66)	48 (73)	46 (61)	
Prior COVID-19^b^	3 (2)	1 (2)	2 (3)	>.99
Positive baseline anti-NP^c^	15 (11)	3 (5)	12 (16)	.05
Dialysis vintage, median (IQR), y	2.65 (1.5-4.6)	2.56 (1.2-4.8)	2.6 (1.6-4.6)	.81
Cause of end-stage kidney disease				
Diabetes	63 (44)	26 (39)	37 (49)	.03
Ischemic nephropathy	27 (19)	8 (12)	19 (25)	
Glomerulonephritis	20 (14)	13 (20)	7 (9)	
Other/unknown	32 (22)	19 (29)	13 (17)	
Comorbidities				
Immunosuppressive treatment^d^	9 (6)	5 (8)	4 (5)	.41
Autoimmune disease	8 (6)	4 (6)	4 (5)	.56
Diabetes	74 (52)	29 (44)	45 (59)	.07
Cancer	23 (16)	12 (18)	11 (14)	.36
Coronary artery disease	53 (37)	22 (33)	31 (41)	.62
Congestive heart failure	37 (26)	15 (23)	22 (29)	.36
Chronic obstructive lung disease	13 (9)	5 (8)	8 (11)	.81
Hypertension	135 (95)	65 (98)	70 (92)	.12
Obesity^e^	10 (7)	2 (3)	8 (11)	.08
Hepatitis B nonresponder^f^	11 (8)	3 (4)	8 (11)	.16

Abbreviations: IQR, interquartile range; NP, nucleocapsid protein.

^a^ A *t* test was used for continuous variables, and χ^2^ or Fisher exact test was used for categorical variables.

^b^ Confirmed using reverse transcriptase polymerase chain reaction.

^c^ Determined by enzyme-linked immunosorbent assays with a threshold for positivity at 0.396. The baseline sample was taken before the first dose in the 1-dose group and before the second dose in the 2-dose group.

^d^ Defined as using any of the following: antimetabolite agent, calcineurin inhibitor, cytotoxic medications, rituximab in previous 6 months, tumor necrosis factor monoclonal antibodies, or glucocorticoids at doses greater than prednisone, 5 mg/d.

^e^ Defined as body mass index greater than 30 (calculated as weight in kilograms divided by height in meters squared).

^f^ Defined as hepatitis B surface antibody less than 10 mIU/mL.

**Figure 1.**
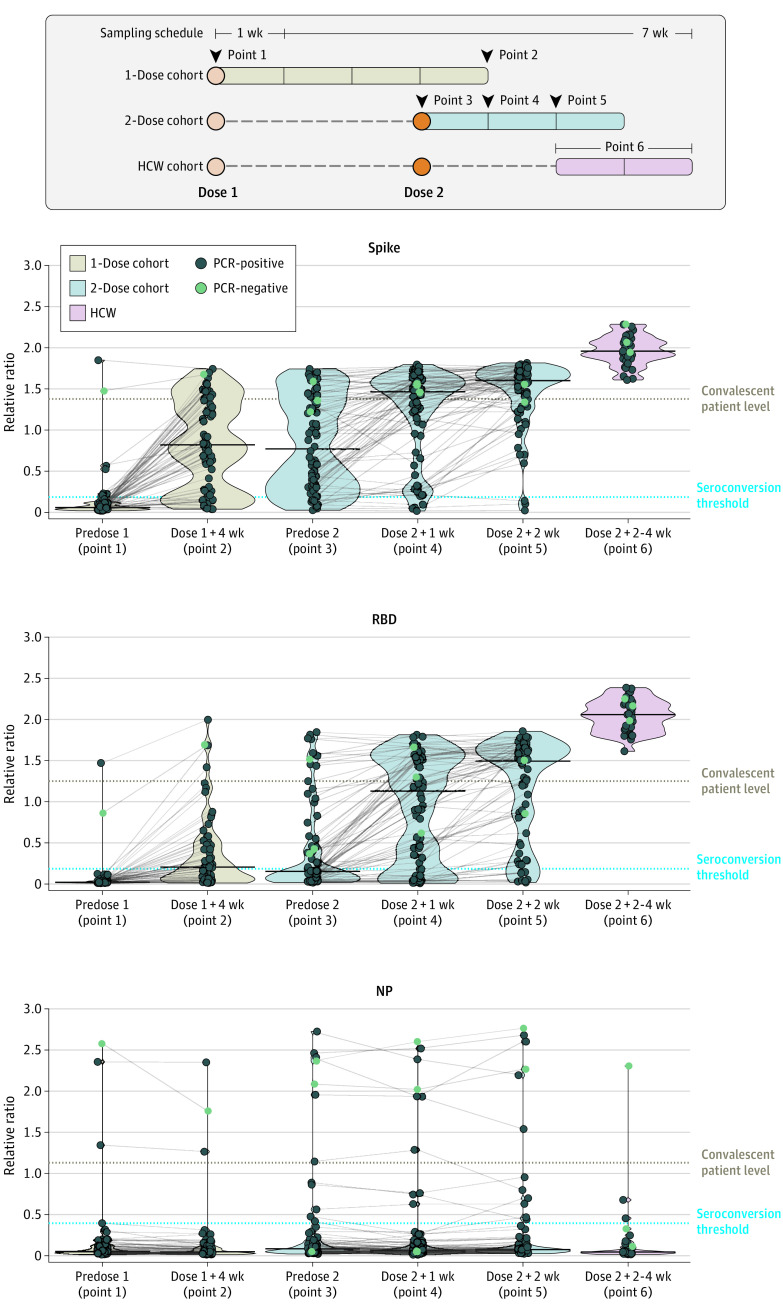
Immunoglobulin G (IgG) Response to Spike, Receptor Binding Domain (RBD), and Nucleocapsid Protein (NP) Antigens of SARS-CoV-2 Following 1 vs 2 Doses of BNT162b2 Vaccine in Patients Receiving Maintenance Hemodialysis HCW indicates health care worker; PCR, polymerase chain reaction.

In 66 patients receiving 1 vaccine dose 4 weeks following the first vaccination, 53 (80%) seroconverted, but only 15 (23%) had anti-spike antibodies exceeding the median relative ratio of convalescent individuals (Table 2). In the 76 patients receiving 2 vaccine doses, the first dose similarly elicited anti-spike seroconversion in 65 patients (86%), with 19 (25%) reaching convalescent levels 28 days postdose. The second vaccine dose, however, induced a more robust response increase, with 43 of 76 patients (57%) reaching convalescent levels by 1 week, further increasing to 52 of 72 patients (72%) by 2 weeks and 69 of 72 patients (96%) reaching seroconversion (Figure 1).

**Table 2.** Rates of Seroconversion and Attaining Convalescent Serum Levels for SARS-CoV-2 IgG Spike, RBD, and NP Antibodies

Study group	Dose^a^	Measurement point	No. (%)	
			Seroconversion^b^	Convalescent serum levels^c^
**Anti-spike**				
Hemodialysis	1 Dose	Predose 1	8/66 (12)	2/66 (3)
		Dose 1 + 4 wk	53/66 (80)	15/66 (23)
	2 Doses	Predose 2	65/76 (86)	19/76 (25)
		Dose 2 + 1 wk	72/76 (95)	43/76 (57)
		Dose 2 + 2 wk	69/72 (96)	52/72 (72)
Health care workers	2 Doses	Dose 2 + 2-4 wk	35/35 (100)	35/35 (100)
**Anti-RBD**				
Hemodialysis	1 Dose	Predose 1	2/66 (3)	1/66 (2)
		Dose 1 + 4 wk	36/66 (55)	4/66 (6)
	2 Doses	Predose 2	31/76 (41)	10/76 (13)
		Dose 2 + 1 wk	58/76 (76)	31/76 (41)
		Dose 2 + 2 wk	63/72 (88)	43/72 (60)
Health care workers	2 Doses	Dose 2 + 2-4 wk	35/35 (100)	35/35 (100)
**Anti-NP**				
Hemodialysis	1 Dose	Predose 1	4/66 (5)	3/66 (5)
		Dose 1 + 4 wk	3/66 (5)	3/66 (5)
	2 Doses	Predose 2	12/76 (16)	7/76 (9)
		Dose 2 + 1 wk	10/76 (13)	7/76 (9)
		Dose 2 + 2 wk	12/72 (17)	6/72 (8)
Health care workers	2 Doses	Dose 2 + 2-4 wk	3/35 (9)	1/35 (3)

Abbreviations: IgG, immunoglobulin G; NP, nucleocapsid protein; RBD, receptor binding domain.

^a^ The second dose was administered 21 days following the first dose.

^b^ Seroconversion threshold represents a positive test and is 0.19 for anti-spike, 0.186 for anti-RBD, and 0.396 for anti-NP antibodies.

^c^ The median level of antigen in convalescent serum is taken 21 to 115 days postsymptom onset is considered a robust antibody response and is 1.38 for anti-spike, 1.25 for anti-RBD, and 1.13 for anti-NP antibodies.

The same overall changes were more pronounced for antibodies to RBD, which are well correlated with neutralizing antibodies.^9,11^ The first vaccine dose elicited a poor anti-RBD response, with seroconversion in 36 of 66 patients (55%) in the 1-dose group and 31 of 76 (41%) in the 2-dose group. After 1 dose, only 4 patients (6%) in the 1-dose group and 10 patients (13%) in the 2-dose group reached convalescent serum levels. However, 1 week after the second dose of vaccine, 58 of 76 (76%) individuals had seroconverted, with 31 of 76 (41%) having antibody levels above the median of convalescent serum. Two weeks after the second dose of vaccine, 63 of 72 patients (88%) seroconverted and 43 of 72 (60%) were above the convalescent serum level. In HCW controls, 100% reached the convalescent levels for both anti-spike and anti-RBD 2 to 4 weeks after 2 doses, and antibody levels in HCWs were significantly higher than in patients receiving hemodialysis at both 1 and 2 weeks after the second dose (anti-spike: 1 week, 53.0; *P* < .001; 2 weeks, 124.0; *P* < .001; anti-RBD: 1 week, 17.0; *P* < .001; 2 weeks, 32.0; *P* < .001; with Mann-Whitney test). Results were similar when individuals with baseline anti-NP seroconversion were excluded (eTable 1, eTable 2, and eFigure in the Supplement).

**Figure 2.**
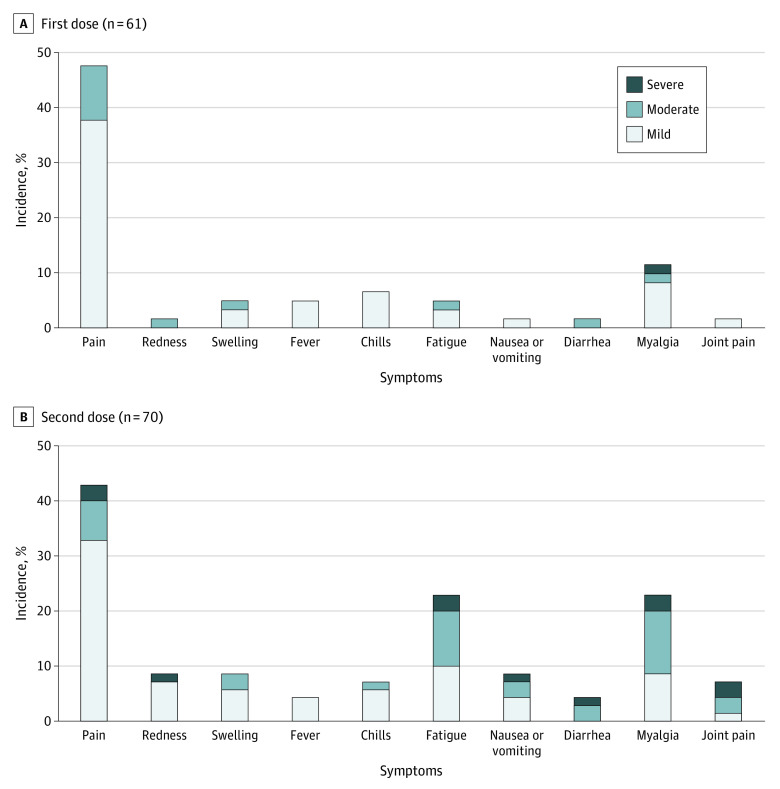
Reactogenicity Rates Following BNT162b2 Vaccine by Symptom Severity Localized and systemic symptoms that occurred after the first (A) and second (B) doses of the vaccine.

The vaccine was generally well tolerated after both the first and second doses (Figure 2). The most common reactions included pain at the injection site, fatigue, and myalgias. The presence of reactogenicity after the second dose was associated with anti-RBD seroconversion (χ^2^ = 12.42; *P* < .001)^2^ but not anti-spike seroconversion. Similarly, multivariate logistic regression found an association between vaccine reactogenicity and anti-RBD seroconversion (odds ratio, 22.90; 95% CI, 2.46-212.83; *P* = .01) but not age or sex (Table 3). Two patients contracted COVID-19 following 2 doses of vaccine despite both having an anti-RBD antibody response above convalescent serum levels before anti-NP seroconversion. Both patients were hospitalized but did not experience severe disease.

**Table 3.** Multivariate Logistic Regression of the Association Between Variables and SARS-CoV-2 Immunogobulin G Anti-RBD Seroconversion or Convalescent Serum Levels 2 Weeks After Second BNT162b2 Dose

Variable	Anti-RBD seroconversion^a^		Anti-RBD reaching median convalescent serum level^b^	
	OR (95% CI)	*P* value	OR (95% CI)	*P* value
Age	1.01 (0.97-1.06)	.58	0.98 (0.94-1.01)	.22
Male sex	1.33 (0.25-7.24)	.74	0.45 (0.16-1.28)	.13
Vaccine reactogenicity^c^	22.86 (2.46-212.83)	.006	1.96 (0.70-5.50)	.20

Abbreviations: OR, odds ratio; IgG, immunoglobulin G; RBD, receptor binding domain.

^a^ Seroconversion threshold represents a positive test and is 0.186 for anti-RBD.

^b^ The median convalescent serum level is taken from COVID-19 survivors 21 to 115 days postsymptom onset and is 1.25 for anti-RBD antibodies.

^c^ Vaccine reactogenicity in patients receiving hemodialysis (n = 70) was binary in the model based on the presence of any of the following symptoms within 14 days after the second vaccine dose: pain, redness, swelling, fever, chills, fatigue, nausea/vomiting, diarrhea, myalgia, and joint pain.

## Discussion

This prospective serologic study found that, although high rates of seroconversion were observed, consistent with other studies in patients receiving hemodialysis,^12^ a robust anti-RBD response defined as reaching convalescent serum levels was seen in less than 10% of patients 28 days after a single vaccine dose. In contrast, 2 weeks after the second dose, 60% of patients receiving hemodialysis had anti-RBD antibody levels comparable with those achieved by patients with COVID-19 infection. Anti-RBD response was lower than anti-spike response, which is of importance because anti-RBD may better associate with viral neutralization.^9^ Comparison of antibody levels with convalescent serum standards from patients with previous COVID-19 infection have been useful comparators for vaccine immunogenicity.^10^ The rationale for using the median convalescent level is that individuals who are vaccinated should at least be expected to reach the antibody levels obtained by individuals with prior COVID-19. We also found that symptoms following the second vaccine dose were associated with anti-RBD seroconversion and may help identify patients who develop some protection.

The response to the second dose, however, was notably weaker than in the HCW controls, all of whom generated robust anti-RBD antibodies. This finding is similar to other high-risk populations. In Canada, patients with cancer and solid organ transplant received 2 doses per manufacturer guidelines because studies demonstrating poor humoral response to 1-dose vaccination led to policy changes.^13,14^ With widespread global vaccine shortages, it is necessary to identify these groups.

### Limitations

This study has limitations. This was a small, single-center study, limiting our ability to fully assess all factors associated with immune response. Follow-up was limited but is ongoing in a larger patient cohort. In addition, the HCW reference population was younger and primarily female. Although our study did not evaluate cell-mediated immunity directly, a good anti-RBD response is required for adequate cell-mediated response.^15^ However, we recognize that longitudinal studies will be required to confirm the clinical significance of comparison with convalescent levels and correlate this outcome with vaccine effectiveness.

## Conclusions

The findings of this study suggest a poor humoral response following a single dose of BNT162b2 COVID-19 vaccine in patients receiving hemodialysis. The second dose should not be delayed in this population.

## Notes

Supplement. **eTable 1.** Comparison of Rates of SARS-CoV-2 IgG Anti-Spike and Anti-RBD Seroconversion in Hemodialysis Patients Receiving 1 Versus 2 Doses of BNT162b2 and HCW Receiving 2 Doses After Including and Excluding Individuals with Baseline Anti-NP Seroconversion **eTable 2.** Rates of Attaining Convalescent Serum Levels for SARS-CoV-2 IgG Anti-Spike and Anti-RBD in Hemodialysis Patients Receiving 1 Versus 2 Doses of BNT162b2 and HCW Receiving 2 Doses After Including and Excluding Individuals with Baseline Anti-NP Seroconversion **eFigure.** SARS-CoV-2 IgG spike, RBD, and NP Antibody Response Following 1 Versus 2 Dose BNT162b2 Vaccine in Hemodialysis Patients Excluding Baseline Anti-NP Seroconversion
