# Errors in Abstract and Figure 1

**DOI:** 10.1001/jamanetworkopen.2022.1422

**Published:** 2022-02-14

In the Original Investigation titled “Evaluation of the SARS-CoV-2 Antibody Response to the BNT162b2 Vaccine in Patients Undergoing Hemodialysis,”^1^ published September 2, 2021, there were errors in the Abstract and Figure 1. In the Abstract Results, the second use of the phrase “in convalescent serum from COVID-19 survivors” should have been omitted. In the Figure 1 color key, the colors given for “PCR-positive” and “PCR-negative” should have been reversed. This article has been corrected.^1^
