# Supplementary material for: Evaluation of the SARS-CoV-2 Antibody Response to the BNT162b2 Vaccine in Patients Undergoing Hemodialysis
Source: JAMA Netw Open. 2021 Sep 2;4(9):e2123622. doi: 10.1001/jamanetworkopen.2021.23622 (PMC8414193; doi:10.1001/jamanetworkopen.2021.23622)
Supplement: Supplement. — eTable 1. Comparison of Rates of SARS-CoV-2 IgG Anti-Spike and Anti-RBD Seroconversion in Hemodialysis Patients Receiving 1 Versus 2 Doses of BNT162b2 and HCW Receiving 2 Doses After Including and Excluding Individuals with Baseline Anti-NP Seroconversion eTable 2. Rates of Attaining Convalescent Serum Levels for SARS-CoV-2 IgG Anti-Spike and Anti-RBD in Hemodialysis Patients Receiving 1 Versus 2 Doses of BNT162b2 and HCW Receiving 2 Doses After Including and Excluding Individuals with Baseline Anti-NP Seroconversion eFigure. SARS-CoV-2 IgG spike, RBD, and NP Antibody Response Following 1 Versus 2 Dose BNT162b2 Vaccine in Hemodialysis Patients Excluding Baseline Anti-NP Seroconversion [file jamanetwopen-e2123622-s001.pdf]

# Supplementary Online Content

Yau K, Abe KT, Naimark D, et al. Evaluation of the SARS-CoV-2 antibody response to the BNT162b2 vaccine in patients undergoing hemodialysis. *JAMA Netw Open*. 2021;4(9):e2123622. doi:10.1001/jamanetworkopen.2021.23622

**eTable 1.** Comparison of Rates of SARS-CoV-2 IgG Anti-Spike and Anti-RBD Seroconversion in Hemodialysis Patients Receiving 1 Versus 2 Doses of BNT162b2 and HCW Receiving 2 Doses After Including and Excluding Individuals with Baseline Anti-NP Seroconversion

**eTable 2.** Rates of Attaining Convalescent Serum Levels for SARS-CoV-2 IgG Anti-Spike and Anti-RBD in Hemodialysis Patients Receiving 1 Versus 2 Doses of BNT162b2 and HCW Receiving 2 Doses After Including and Excluding Individuals with Baseline Anti-NP Seroconversion

**eFigure.** SARS-CoV-2 IgG spike, RBD, and NP Antibody Response Following 1 Versus 2 Dose BNT162b2 Vaccine in Hemodialysis Patients Excluding Baseline Anti-NP Seroconversion

This supplementary material has been provided by the authors to give readers additional information about their work.

© 2021 Yau K et al. *JAMA Network Open.*

**eTable 1. Comparison of Rates of SARS-CoV-2 IgG Anti-Spike and Anti-RBD Seroconversion in Hemodialysis Patients Receiving 1 Versus 2 Doses of BNT162b2 and HCW Receiving 2 Doses After Including and Excluding Individuals with Baseline Anti-NP Seroconversion**

| Anti body | Study group | | Time point | Seroconversion No. (%)[a] | |
|---|---|---|---|---|---|
| | | | | Including baseline anti-NP seroconversion | Excluding baseline anti-NP seroconversion |
| Anti-spike | HD | 1st | Pre-dose one | 8/66 (12%) | 6/63 (10%) |
| | | | Dose one + 4 weeks | 53/66 (80%) | 50/63 (79%) |
| | | 2nd[b] | Pre-dose two | 65/76 (86%) | 53/64 (83%) |
| | | | Dose two + 1 week | 72/76 (95%) | 60/64 (94%) |
| | | | Dose two + 2 weeks | 69/72 (96%) | 57/60 (95%) |
| | HCW | 2nd[b] | Dose two + 2-4 weeks | 35/35 (100%) | 35/35 (100%) |
| Anti-RBD | HD | 1st | Pre-dose one | 2/66 (3%) | 0/63 (0%) |
| | | | Dose one + 4 weeks | 36/66 (55%) | 33/63 (52%) |
| | | 2nd[b] | Pre-dose two | 31/76 (41%) | 22/64 (34%) |
| | | | Dose two + 1 week | 58/76 (76%) | 47/64 (73%) |
| | | | Dose two + 2 weeks | 63/72 (88%) | 51/60 (85%) |
| | HCW | 2nd[b] | Dose two + 2-4 weeks | 35/35 (100%) | 35/35 (100% |

Abbreviations: HD, hemodialysis, HCW, health care worker; NP, nucleocapsid protein; RBD, receptor binding domain.
[a]Seroconversion threshold represents a positive test and are 0.19, 0.186, 0.396 for anti-spike, anti-RBD, and anti-NP respectively.
[b]Second dose was administered 21 days following first dose.

© 2021 Yau K et al. *JAMA Network Open.*

## eTable 2. Rates of Attaining Convalescent Serum Levels for SARS-CoV-2 IgG Anti-Spike and Anti-RBD in Hemodialysis Patients Receiving One Versus Two Doses of BNT162b2 and HCW Receiving Two Doses After Including and Excluding Individuals with Baseline Anti-NP Seroconversion

| Anti body | Study group | | Time point | Convalescent serum levels No. (%)[a] | |
|---|---|---|---|---|---|
| | | | | Including baseline anti-NP seroconversion | Excluding baseline anti-NP seroconversion |
| Anti-spike | HD | 1st | Pre-dose one | 2/66 (3%) | 0/63 (0%) |
| | | | Dose one + 4 weeks | 15/66 (23%) | 13/63 (21%) |
| | | 2nd[b] | Pre-dose two | 19/76 (25%) | 14/64 (22%) |
| | | | Dose two + 1 week | 43/76 (57%) | 34/64 (53%) |
| | | | Dose two + 2 weeks | 52/72 (72%) | 43/60 (72%) |
| | HCW | 2nd[b] | Dose two + 2-4 weeks | 35/35 (100%) | 35/35 (100%) |
| Anti-RBD | HD | 1st | Pre-dose one | 1/66 (2%) | 0/63 (0%) |
| | | | Dose one + 4 weeks | 4/66 (6%) | 2/63 (3%) |
| | | 2nd[b] | Pre-dose two | 10/76 (13%) | 7/64 (11%) |
| | | | Dose two + 1 week | 31/76 (41%) | 25/64 (39%) |
| | | | Dose two + 2 weeks | 43/72 (60%) | 35/60 (58%) |
| | HCW | 2nd[b] | Dose two + 2-4 weeks | 35/35 (100%) | 35/35 (100%) |

Abbreviations: HD, hemodialysis, HCW, health care worker; NP, nucleocapsid protein; RBD, receptor binding domain.
[a]The median level of antigen in convalescent serum taken 21-115 days post-symptom onset is considered a robust antibody response and are 1.38,1.25, and 1.13 for anti-spike, anti-RBD, and anti-NP antibodies respectively.
[b]Second dose was administered 21 days following first dose.

© 2021 Yau K et al. *JAMA Network Open.*

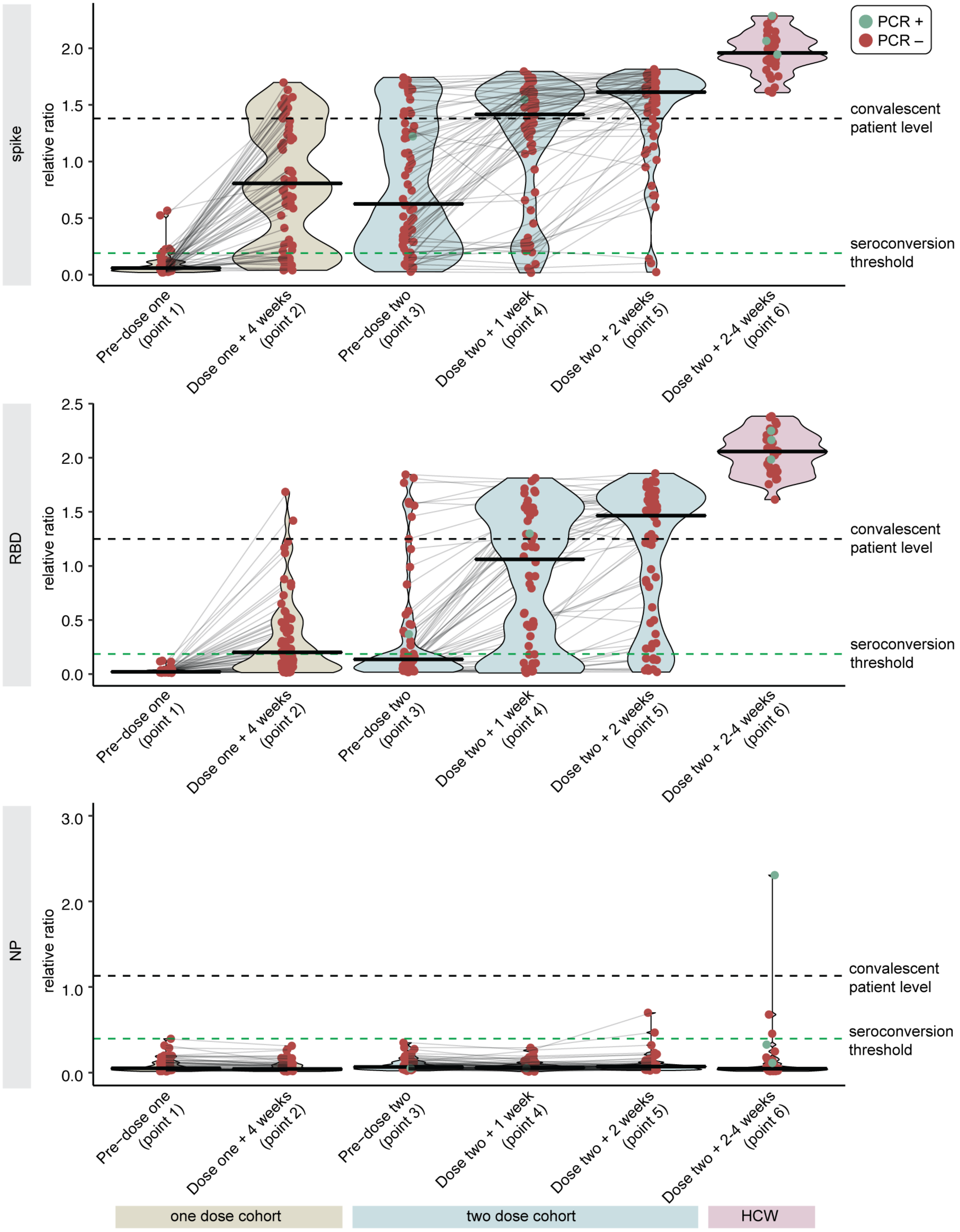


© 2021 Yau K et al. *JAMA Network Open.*

**eFigure: SARS-CoV-2 IgG spike, RBD, and NP Antibody Response Following One Versus Two Dose BNT162b2 Vaccine in Hemodialysis Patients Excluding Baseline Anti-NP Seroconversion.**

Antibody levels are reported as relative ratios to synthetic standards. The samples are grouped into two cohorts who received one (n=63) or two doses (n=64) of vaccine after excluding individuals with Anti-NP seroconversion at baseline. Dots represent individual serum samples collected at the indicated times, and the samples from the same patients are connected by lines. Green dots indicate individuals with prior RT-PCR confirmed SARS-CoV-2 infection. Seroconversion threshold represents a positive test and are 0.19, 0.186, and 0.396 for anti-spike, anti-RDB, and anti-NP antibodies respectively. The median level of antigen in convalescent serum taken 21-115 days post-symptom onset is considered a robust antibody response and is 1.38, 1.25, and 1.13 for anti-spike, anti-RBD, and anti-NP antibodies respectively. Healthy control workers (n=35) received two doses of vaccines with serologic measurement 2-4 weeks following dose 2. Abbreviations: HCW, health care worker; NP, nucleocapsid protein; RBD, receptor binding domain.

© 2021 Yau K et al. *JAMA Network Open.*
